# Perspectives on death and dying: a study of resident comfort with End-of-life care

**DOI:** 10.1186/s12909-016-0819-6

**Published:** 2016-11-21

**Authors:** Jessica M. Schmit, Lynne E. Meyer, Jennifer M. Duff, Yunfeng Dai, Fei Zou, Julia L. Close

**Affiliations:** 1 Department of Medicine, Division of Hematology and Oncology, University of Florida, 1600 SW Archer Rd, 32610 Gainesville, FL USA; 2 College of Medicine, Office of Graduate Medical Education, University of Florida, Gainesville, FL USA; 3 Department of Biostatistics, University of Florida, Gainesville, FL USA

**Keywords:** Graduate Medical Education, End of life care, Terminal Care, Internship and Residency, Palliative Care

## Abstract

**Background:**

Despite the benefits to early palliative care in the treatment of terminal illness, barriers to timely hospice referrals exist. Physicians who are more comfortable having end-of-life (EOL) conversations are more likely to refer to hospice. However, very little is known about what factors influence comfort with EOL care.

**Methods:**

An anonymous survey was sent to all the residents and fellows at a single institution. Self-reported education, experience and comfort with EOL care was assessed. Using multivariate logistic regression analysis, variables that influenced comfort with EOL conversations were analyzed.

**Results:**

Most residents (88.1%) reported little to no classroom training on EOL care during residency. EOL conversations during residency were frequent (50.6% reported > 10) and mostly unsupervised (61.9%). In contrast, EOL conversations during medical school were infrequent (3.7% reported >10) and mostly supervised (78.6%). Most (54.3%) reported little to no classroom training on EOL care during medical school. Physicians that reported receiving education on EOL conversations during residency and those who had frequent EOL conversations during residency had significantly higher comfort levels having EOL conversations (*p* = 0.017 and *p* = 0.003, respectively). Likewise, residents that felt adequately prepared to have EOL conversations when graduating from medical school were more likely to feel comfortable (*p* = 0.030).

**Conclusions:**

Most residents had inadequate education in EOL conversation skills during medical school and residency. Despite the lack of training, EOL conversations during residency are common and often unsupervised. Those who reported more classroom training during residency on EOL skills had greater comfort with EOL conversations. Training programs should provide palliative care education to all physicians during residency and fellowship, especially for those specialties that are most likely to encounter patients with advanced terminal disease.

**Electronic supplementary material:**

The online version of this article (doi:10.1186/s12909-016-0819-6) contains supplementary material, which is available to authorized users.

## Background

Studies have shown the benefits of involving palliative care early in the care of patients with terminal disease. Implementation of palliative care in addition to usual care improves patient survival, [1–3] decreases health care costs, [4–6] and improves patients’ and caregivers’ quality of life (QOL) [2, 7–10]. Despite this, hospice and palliative care services are underutilized, [11] with studies demonstrating that up to 50–55% of terminally ill, hospice eligible patients are never referred by their treating physician [12, 13].

There are many barriers to appropriate and timely hospice referrals, arising from the patient, health care system or physicians [14]. Patients and their caregivers may have difficulty accepting the illness as terminal. They may also fear hospice enrollment or may not understand the benefits to hospice care [7, 15, 16]. Patient age, gender and race are known to influence hospice utilization [17–20]. Furthermore, the health care system has often limited hospice eligibility to those with an estimated life-expectancy of 6 months or less, forcing patients to choose between a curative versus hospice approach to their care [16, 21]. Physicians often delay discussing hospice and palliative care until late in the disease course [22, 23]. They may assume that patients or their families do not wish to know about hospice or are unwilling to accept it [12, 24–26]. They also may have misconceptions about eligibility criteria or services offered by hospice [12, 16, 21]. Lastly, physicians may delay discussing hospice and palliative care as to avoid uncomfortable conversations and emotions [26, 27].

Although much is known about potential barriers to hospice referrals, very little is known about what determines physician comfort with end-of-life (EOL) conversations and dying patients. A study by Kogan et al. demonstrated that physicians who are comfortable discussing end-of-life care issues are more likely to refer to hospice [28]. By identifying what factors influence physician comfort, it may be possible to implement changes that can lead to more timely hospice referrals, less-aggressive care at the EOL and better QOL for patients. In the present study, we aimed to better understand the factors that may influence physician comfort with EOL care. We hypothesized that physician demographics, spirituality and education/experience may all play a role in comfort with EOL care.

## Methods

In February 2015, a survey was sent electronically to all the residents and fellows (*n* = 787) at a single academic institution. They were asked to participate in a survey assessing their comfort with caring for dying patients and EOL issues. A copy of the informed consent was provided along with a link to the survey. Participation was voluntary and results were anonymous. The study design, survey questions, and consent process were approved by the Institutional Review Board. The online survey consisted of a total of 33 questions (see Additional file 1 for a full list of questions and answer choices) and was conducted using Qualtrics™ software. Statistical analysis was performed using SPSS version 22 and SAS version 9.4 software.

To assess comfort, participants were queried as to how comfortable they felt having EOL conversations with patients and families using a five-point Likert scale with choices ranging from “very uncomfortable” to “very comfortable”. Answer choices were reverse coded for analysis with a score of 5 being most comfortable and 1 being least comfortable.

To assess pairwise relationship between each pair of collected variables, we used 1) Spearman’s rank correlation coefficient for all questions with ranked variable pairs, 2) the Mann-Whitney test or the Kruskal-Wallis test for ranked variable and nominal variable pairs, and 3) a chi-square test or Fisher’s exact test for all non-ranked variable pairs. Logistic regression analysis was used to independently evaluate the relationship between the dependent variable, comfort with EOL conversations, and a set of confounding variables. Response choices for the dependent variable were combined into three groups for the analysis (1–2 uncomfortable; 3 neutral; 4–5 comfortable). Questions left blank and answer choices “not applicable”, “not sure”, or “prefer not to say” were excluded from the analysis. To test for potential non-responder bias, we performed logistic regression analysis on responders versus non-responders using two known variables, department and gender, as covariates.

Of the original 33 survey questions, 21 were included in the multivariate logistic regression analysis. Reasons for exclusion from the analysis included: low response rate (*n* = 5), multiple selected combinations unable to be grouped (*n* = 4), highly correlated (*p* < 0.0001) with another question (*n* = 2), and descriptive text (*n* = 1). Of the questions excluded due to low response, all were follow-up questions, visible only to a subset of participants based on their previous answer choices. The final model was selected using stepwise selection procedure with 0.1 as the entry and stay probabilities for each variable. Only surveys with all answered questions were included in the stepwise selection procedure (*n* = 101).

## Results

Of the 787 residents and fellows, 175 (22.2%) participated in our voluntary survey. Residents from all 18 departmental specialties responded to our survey. There were more male participants (58.9%) than female, which is consistent with our institutional demographics. The typical participant was Caucasian (72.1%), US born (78.9%) and between the ages of 25–34 (84.2%). Approximately half identified themselves as Christian (50.7%). Full demographic data is listed in Table 1. When testing for non-responder bias, no bias was identified for gender (*p* = 0.608), however, bias was found when looking at department (*p* = 0.003), suggesting some participants were more or less likely to respond to our survey based on their department of training.

**Table 1:** Demographics^a^

Variable	Number (%)
Age in years	
20–24	1 (0.7%)
25–29	42 (28.8%)
30–34	81 (55.5%)
35–39	15 (10.3%)
40+	7 (4.8%)
Gender	
Male	86 (58.9%)
Female	60 (41.1%)
Race/Ethnicity	
Caucasian/White	106 (72.1%)
Black/African American	3 (2.0%)
Hispanic/Latino	8 (5.4%)
Asian	26 (17.7%)
Pacific Islander/Hawaiian	2 (1.4%)
Native American/Native Alaskan	0 (0.0%)
Multiracial	2 (1.4%)
Religious Affiliation	
Christian	74 (50.7%)
Muslim	5 (3.4%)
Hindu	11 (7.5%)
Buddhist	0 (0%)
Jewish	5 (3.4%)
Atheist	8 (5.5%)
Agnostic	18 (12.3%)
Other	4 (2.7%)
Spiritual, but not religious	27 (18.5%)
None	5 (3.4%)
Place of birth	
U.S.	116 (78.9%)
Non-U.S.	31 (21.1%)
Department	
Internal Medicine	35 (27.1%)
Surgery	15 (11.6%)
Pediatrics	12 (9.3%)
Anesthesiology	12 (9.3%)
Family Medicine	9 (7.0%)
Radiology	7 (5.4%)
Neurosurgery	5 (3.9%)
Radiation Oncology	4 (3.1%)
Ophthalmology	4 (3.1%)
Obstetrics and Gynecology	4 (3.1%)
Pathology	4 (3.1%)
Psychiatry	4 (3.1%)
Urology	4 (3.1%)
Orthopedic Surgery	3 (2.3%)
Emergency Medicine	2 (1.6%)
Neurology	2 (1.6%)
Dermatology	2 (1.6%)
Otolaryngology	1 (0.8%)
Post-graduate year	
PGY1-2	50 (35.7%)
PGY 3-4	50 (35.7%)
PGY 5+	40 (28.6%)
Type of Medical School	
Allopathic (MD)	113 (77.9%)
Osteopathic (DO)	8 (5.5%)
International	24 (16.6%)

^a^Missing variables not included

Comfort with EOL conversations was gauged by asking the question, “At your current level of training, do you feel comfortable having end-of-life discussions with patients/families on your own?” (Table 2). The average comfort level having EOL conversations on a 5-point Likert scale was 3.90. The majority of participants (74.5%) felt comfortable having EOL discussions. Only the minority were neutral (11.7%) or uncomfortable (13.8%) having these conversations.

**Table 2:** Comfort Question

Comfort with EOL discussions *n* = 145	
“At your current level of training, do you feel comfortable having end-of-life discussions with patients/families on your own?”	
Answer choices:^a^	Number(%):
5 = I feel very comfortable	47 (32.4%)
4 = I feel mostly comfortable	61 (42.1%)
3 = I am neither comfortable nor uncomfortable	17 (11.7%)
2 = I am mostly uncomfortable	16 (11.0%)
1 = I feel very uncomfortable	4 (2.8%)

^a^Answer choices “I’m not sure” and “other” were excluded from analysis

We were very interested in the amount of formal education residents received on having EOL discussions. We asked participants to estimate the amount of time they spent in classroom training on having EOL discussions during medical school and residency (Table 3). Answer choices were: “None”, “Very little (1–2 lectures)”, “Some (1-2 week course or lecture series)”, or “A Lot (>3 weeks)”. We found that most residents reported very little to no training on EOL discussions in both medical school and residency (54.3% and 88.1%, respectively). Only 45.7% of participants reported an adequate amount (“some” or “a lot”) of EOL training during medical school; whereas this number fell to only 11.9% during residency.

**Table 3:** Education and Experience

Question	Number (%)
Classroom training on EOL in medical school	
None	14 (8.6%)
Very Little	75 (46.0%)
Some	64 (39.3%)
A lot	10 (6.1%)
Classroom training on EOL in residency	
None	59 (39.1%)
Very Little	74 (49.0%)
Some	17 (11.3%)
A lot	1 (0.7%)
Number of EOL conversations in medical School	
None	53 (33.1%)
1–5	83 (51.9%)
6–10	18 (11.3%)
11–15	5 (3.1%)
16–25	0
26–50	1 (0.6%)
> 50	0
Number of EOL conversations in residency	
None	10 (6.7%)
1–5	41 (27.3%)
6–10	23 (15.3%)
11–15	21 (14.0%)
16–25	21 (14.0%)
26–50	17 (11.3%)
> 50	17 (11.3%)
EOL supervision in medical school	
Always	50 (51.0%)
Mostly supervised	27 (27.6%)
Mostly unsupervised	15 (15.3%)
Never	6 (6.1%)
EOL supervision in residency	
Always	13 (9.4%)
Mostly supervised	40 (28.8%)
Mostly unsupervised	58 (41.7%)
Never	28 (20.1%)
Not at all	32 (22.5%)
Felt prepared for EOL after medical school	
Not at all	25 (16.1%)
A little	59 (38.1%)
Somewhat	50 (32.3%)
Very	16 (10.3%)
Fully	5 (3.2%)

We asked participants to estimate the number of times they had an EOL conversation during medical school and residency (Table 3). EOL conversations during medical school were relatively uncommon. A third of participants (33.1%) had never had an EOL conversation during medical school and only 3.7% reported having more than ten EOL conversations during medical school. The frequency of EOL conversations during residency increased; 50.6% reported more than ten conversations and only 6.7% reported never having had an EOL conversation.

Finally, participants were asked how much supervision they received while having EOL conversations with patients and their families (Table 3). Most (77.6%) reported they were “always” or “mostly” supervised during medical school, however this fell to 38.1% during residency with the majority of residents (61.9%) reporting their EOL conversations were “mostly unsupervised” or “never supervised”.

To assess which factors may influence resident comfort with EOL conversations, we performed a multivariate logistic regression analysis. The questions analyzed fell into three categories: demographics, education/experience, and spirituality. All of the demographic variables (Table 1), including age, gender, race/ethnicity, religious affiliation, place of birth, department, post-graduate year and type of medical school, were included in the logistic regression; however, none of these were shown to influence physician comfort with EOL conversations. The questions relating to spirituality included belief in an afterlife (*n* = 1), fear of death (*n* = 1), and metaphysical experiences (*n* = 2). Similarly, none of these variables were found to correlate with resident comfort having EOL conversations.

In the education/experience category, we included all of the questions listed in Table 2 except supervision in medical school (eliminated due to low numbers). We did not find any statistically significant associations with classroom training during medical school, number of EOL conversations during medical school, or EOL supervision in residency. Three variables in the education/experience domain were found to have a strong association with comfort having EOL conversations and are listed in Table 4. These include the amount of classroom training on EOL discussions during residency (OR = 3.3 (95% CI: 1.2–8.9); *p* = 0.017), the number of EOL conversations during residency (OR = 2.1 (95% CI: 1.3–3.4); *p* = 0.003) and also how prepared the resident felt to have EOL conversations when graduating from medical school (OR = 2.1 (95% CI: 1.1–4.1); *p* = 0.030). Residents who reported greater classroom time on EOL care, had more EOL discussions, or felt more prepared to have EOL discussions when graduating from medical school were significantly more likely to feel comfortable having EOL discussions.

**Table 4:** Logistic regression analysis^a^

Variable:	Comfortable with EOL conversations^b^
Increased classroom training in residency on EOL skills	OR = 3.3 (95% CI: 1.2–8.9); *p* = 0.017
Felt more prepared after medical school to have EOL conversations	OR = 2.1 (95% CI: 1.1–4.1); *p* = 0.030
Higher numbers of EOL conversations in residency	OR = 2.1 (95% CI: 1.3–3.4); *p* = 0.003

^a^Only variables found to have statistically significant association with comfort are shown

^b^ *OR* odds ratio, *CI* confidence interval

## Discussion

Previous studies have shown that most physicians receive very little palliative care and EOL training during medical school and residency. In a study by Van Aalst-Cohen, et al., only 30% of medical schools required education on hospice and palliative care [29]. Despite the recommendation by the Institute of Medicine that “educational institutions and professional societies provide training in palliative care domains throughout the professional’s career,” palliative care education remains underrepresented in medical school and residency curricula [11]. In the present study, over half of residents reported inadequate EOL education during medical school and nearly 90% reported inadequate education during residency. Although the low rate of EOL education in residency may be specific to our institution, the low rates in medical school likely reflect a global problem of EOL care receiving little attention.

EOL conversations have been described as a medical procedure or skill [30]. Further supporting this idea, advanced care planning has recently been added as a billable procedure using current procedural terminology (CPT) codes. When viewed in this way, it is easy to understand why adequate training, experience and supervision are essential when learning how to have effective and timely EOL conversations. In this study, we demonstrate that many doctors graduate from medical school with very little experience or training having EOL conversations. During residency, these doctors receive very little additional preparation, yet are asked to lead EOL conversations often and unsupervised. Poor EOL conversation skills can strain the doctor-patient relationship, lead to unnecessary tests and procedures and result in higher healthcare costs and discomfort for patients.

EOL conversation skills can be taught. Multiple studies have demonstrated that EOL conversation training programs can be developed and implemented for medical students and residents and lead to improved conversation skills [31–34]. A recent meta-analysis published by Chung et al. found that educational interventions to improve EOL communication were associated with greater self-efficacy, more knowledge and improved communication scores [35]. Our findings are consistent with previous work and suggest that modifying educational programs to place greater emphasis on EOL care and communication can lead to greater comfort and skill in these areas.

Of the three domains we investigated as possibly influencing comfort with EOL care—physician demographics, spirituality and education/experience—we were only able to demonstrate a relationship between physician education/experience and comfort with EOL care. Previous studies have suggested that demographic variables such as physician race, age and specialty may influence hospice referral patterns [12, 28, 36]. The relatively young age, lack of minority representation and small sample size of our study may have caused small differences to be missed. Likewise, previous studies have shown that physician religion and spirituality may also influence care, [37–39] although this was not appreciated in the present study. More research on the influence of physician attitudes and beliefs on patient care is needed.

Limitations of this study are inherent in its design as a survey. There may be recall bias as accurate assessment of classroom training, number of EOL conversations and degree of supervision relies on memory and may not be reflective of the actual experience of these resident physicians. This survey was conducted at a single institution and only included residents/fellows in training, and therefore may not be generalizable to all institutions or all physicians or residents. As noted previously, we did find bias with some departments being over- and underrepresented in our study. Residents were aware of the nature of the survey prior to participating, which may have led to selection bias towards physicians interested in this topic. The survey was voluntary and our response rate was low (22.2%), which also threatens the validity of the study.

Another limitation in the study was that we did not confirm the amount of EOL education that was actually given to residents in the various departments. It is possible that those who feel uncomfortable with EOL conversations are receiving the same amount of education, but are less likely to recall the lectures they received. Although we know from prior studies that comfort with EOL care correlates with hospice referral among physicians, this was not addressed in the present study. Additional research is needed to investigate how palliative care educational programs may influence the long-term practice patterns of physicians.

## Conclusion

Resident education in EOL care is associated with enhanced comfort with EOL conversations. By increasing the amount of formal training and supervision, especially during residency, physician comfort may improve, leading to earlier and more appropriate hospice referrals, better end-of-life care and improved quality-of-life for our terminal patients. Palliative care and EOL discussions should be included as an additional communication milestone when the next iteration of the Milestone Project by the Accreditations Council for Graduate Medical Education (ACGME) and American Board of Medical Specialties (ABMS) specialty boards is developed.

## Additional file

Additional file 1: Physician Perspectives on Death and Dying. (DOCX 20 kb)
