# Supplementary material for: Perspectives on death and dying: a study of resident comfort with End-of-life care
Source: BMC Med Educ. 2016 Nov 21;16:297. doi: 10.1186/s12909-016-0819-6 (PMC5117582; doi:10.1186/s12909-016-0819-6)
Supplement: Additional file 1: — Physician Perspectives on Death and Dying. (DOCX 20 kb) [file 12909_2016_819_MOESM1_ESM.docx]

Additional file 1: Physician Perspectives on Death and Dying

    Thank you for taking this survey.   There is a minimal risk that security of any online data may be breached.  Since the online host uses both encryption and firewalls, and your data will be downloaded from the server soon after you complete the survey, it is unlikely that a security breach of the online data will result in any adverse consequence for you.  For further information, you may visit the links below:   Qualtrics Privacy Statement:  http://www.qualtrics.com/privacy-statement/ Qualtrics Security Statement:  http://www.qualtrics.com/security-statement/

How much classroom training did you receive in medical school on having end-of-life discussions and discussing death/dying with patients?

- None
- Very little (1-2 lectures)
- Some (1-2 week course or lecture series)
- A Lot (>3 weeks total)
- I don't know

To the best of your knowledge, approximately how many times during medical school have you had to have an end-of-life discussion with a patient or family?  Examples include: telling a patient they are dying, recommending a transition to hospice/palliative care, discussing withdrawal of care in a patient unlikely to survive, calling family and reporting the death of their loved one, etc.

- None
- 1-5 times
- 6-10
- 11-15
- 16-25
- 26-50
- >50 times

Did you receive supervision during these end-of-life conversations by an attending or supervising resident?

- Yes, I always had supervision
- Mostly supervised (>50% of time)
- Mostly unsupervised (
- No, I never had supervision
- I'm not sure

During medical school, how often did you feel that a lack of adequate supervision or training for end-of-life discussions negatively impacted your patients care?

- Always
- Often
- Sometimes
- Rarely
- Never
- I'm not sure

When graduating from medical school did you feel prepared to have end-of-life discussions with your terminal patients or their families?

- Not at all prepared
- A little prepared
- Somewhat prepared
- Very prepared
- Fully prepared

During residency, how much classroom training did you receive on having end-of-life discussions with patients?

- None
- Very little (1-2 lectures)
- Some (1-2 week course or lecture series)
- A Lot (>3 weeks)
- I don't know

How many times during residency have you had to have an end-of-life discussion with a patient or family?

- None
- 1-5 times
- 6-10
- 11-15
- 16-25
- 26-50
- >50

Did you receive supervision during these conversations by an attending or supervising resident?

- Yes, I always had supervision
- Mostly supervised (>50% of time)
- Mostly unsupervised (
- No, I never had supervision
- I'm not sure

During residency, did you ever feel that a lack of adequate supervision or training for end-of-life discussions negatively impacted your patients care?

- Yes, always
- Often
- Sometimes
- Rarely
- Never
- I'm not sure

At your current level of training, do you feel comfortable having end-of-life discussions with patients/families on your own?

- Yes, I feel very comfortable
- I feel mostly comfortable
- I am neither comfortable nor uncomfortable
- I feel mostly uncomfortable
- I feel very uncomfortable
- I'm not sure

Please pick your comfort level with taking care of dying patients: (choose which statement best reflects you)

- I am very uncomfortable with these patients and try to avoid them
- I am somewhat uncomfortable and prefer not to take care of them
- I am neutral and do not mind taking care of them
- I am mostly comfortable and find some satisfaction taking care of them
- I am very comfortable and find great satisfaction taking care of them
- I'm not sure
- Other: (please describe) ____________________

Do you ever find yourself being negatively affected by working with terminal patients: (Example: feeling sad during or after work)

- Always
- A lot
- Some
- A little
- Not at all
- I'm not sure

Personal feelings about death/dying: (please choose which statement best reflects you)

- I am extremely afraid of dying
- I am very afraid of dying
- I am moderately afraid of dying
- I am a little afraid of dying
- I have no fear of dying
- I'm not sure

Please select if you have experienced the death of any of the following people in your life:  (select all that apply)

- Parent
- Grandparent
- Spouse
- Child
- Brother/Sister
- Cousin
- Great-grandparent
- Aunt/Uncle
- Close friend
- Boyfriend/Girlfriend
- other ____________________
- None

What is your age?

- 20-24
- 25-29
- 30-34
- 35-39
- 40+

What is your gender?

- Male
- Female

Racial/ethnic background?

- Caucasian/White
- Black/African American
- Hispanic/Latino
- Asian
- Native American/Native Alaskan
- Pacific Islander/Hawaiian
- Multiracial ____________________
- I prefer not to say

Place of birth?

- United States
- Non-US

Childhood religion (how were you raised)? (check all that apply)

- Christian
- Muslim
- Hindu
- Buddhist
- Jewish
- Atheist (the belief that God does not exist)
- Agnostic (the belief that we cannot know if God exists or not)
- Other, not listed ____________________
- Spiritual, but not religious
- None
- Prefer not to say

Current religious affiliation or beliefs: (check all that apply)

- Christian
- Muslim
- Hindu
- Buddhist
- Jewish
- Atheist (the belief that God does not exist)
- Agnostic (the belief that we cannot know if God exists or not)
- Other, not listed ____________________
- Spiritual, but not religious
- None
- Prefer not to say

Type of Medical School?

- US Allopathic Medical School (MD) or Canadian Medical School
- US Osteopathic Medical School (DO)
- International Medical School
- Other ____________________

What department of training are you currently in? (you may choose "prefer not to say")

- Anesthesiology
- Dermatology
- Emergency Medicine
- Family Medicine
- Internal Medicine
- Neurology
- Neurosurgery
- Obstetrics and Gynecology
- Ophthalmology
- Orthopedic Surgery
- Otolaryngology
- Pathology
- Pediatrics
- Psychiatry
- Radiology
- Radiation Oncology
- Surgery
- Urology
- Prefer not to say

What is your current post-graduate year level of training?

- PGY1-2
- PGY3-4
- PGY5+
- Prefer not to say

This next question set pertains to the metaphysical domain.  Metaphysics is a branch of philosophy that encompasses the study of a reality beyond what is perceptible to the senses.    Metaphysical or transcendental experiences are those that do not seem to have a scientific or physical explanation.  "Meta" = beyond; "physics" = physical.  Examples of modern-day metaphysical experiences would include seeing visions, visitations from deceases relatives, seeing angels or ghosts, having an out-of-body experience, near-death experience, etc.

Has a patient ever reported to you that they had a metaphysical experience?

- Yes
- No
- I'm not sure

In your opinion, how often are these experiences caused by biochemical disturbances? (e.g. delirium, illusions, hallucinations, drugs, hypoxia, mental illness, etc)

- Always
- Most of the time
- Sometimes
- Rarely
- Never
- I'm not sure

Has a family member or friend ever reported to you that they had a metaphysical experience?

- Yes
- No
- I'm not sure

In your opinion, how often were your friends' or family members' expreience(s) caused by biochemical disturbances? (e.g. delirium, illusions, hallucinations, drugs, hypoxia, mental illness, etc.)

- Always
- Most of the Time
- Sometimes
- Very rarely
- Never
- I'm not sure

Have you personally had an experience you would consider metaphysical in nature?

- Yes
- No
- Maybe
- I'm not sure
- I prefer not to say

Have you ever told anyone about this experience?

- Yes, I talk about this openly
- Yes, but only with a few of my closest friends/family
- No, I have never shared this
- I'm not sure or cannot remember

What stops you from discussing this experience openly with others? (select all that apply)

- I'm not sure if it was real
- I'm afraid of what people will think of me
- I feel it is private
- I think people will think I'm crazy
- I do not think people will believe me
- I have discussed it in the past and gotten a negative reaction
- Other ____________________

If comfortable, please share the nature of this experience.

Do you believe in an afterlife? (the notion that an intrinsic part of who you are continues to exist after your physical death)

- Definitely yes
- Probably yes
- Undecided
- Probably not
- Definitely not
- I prefer not to say

Please select the statement(s) that most accurately reflect your beliefs about metaphysical experiences: (select all that apply)

- I believe they are real glimpses into a reality beyond normal human perception
- I believe they are imaginations of the mind and not based in reality
- I believe some are probably real and others are not
- I believe most people who report such things are crazy or delusioned
- I believe most people who report such things are lying
- I am not sure
- Other (please explain) ____________________
